# Self- Reported Personal and Family History of Cancers in Brunei Darussalam: Result of an Integrated Health Survey

**DOI:** 10.31557/APJCP.2019.20.11.3279

**Published:** 2019

**Authors:** Vui Heng Chong, Chong Kadir, Zakaria Kamis, Norhayati Kassim, Chee Fui Chong

**Affiliations:** 1 *Department of Medicine, *; 3 *Department of Surgery, RIPAS Hospital 2-PAPRSB Institute of Health Science, Universiti Brunei Darussalam, *; 2 *Health Promotion Centre, Minsitry of Health, Brunei Darussalam. *

**Keywords:** Neoplasms, prevalence, cancers, malignancy, family history

## Abstract

**Introduction:**

Cancers remain an important cause of mortality and morbidity, and overall incidence of cancers continues to increase worldwide with some cancers increasing while others decreasing. Understanding the epidemiology of cancer burden is important for health care planning. Most studies to date have reported incidence based on cancer registry. This aim of this study is to report the incidence of self-reported personal and family history of cancers.

**Materials and Methods:**

Data on cancers were extracted from an anonymized database of a survey (Integrated Health Screening Survey) for civil servants conducted between 2008 and 2013 (N=21,437, mean age 40.61 ± 9.46 years old, men 45.1%).

**Results:**

The overall incidence of self-reported cancers was 11.2%; personal and family histories were 0.6% and 9.4% respectively (1.2% did not state if cancers were either personal or family history). Commonly self-reported personal history of cancers were cancer of the breast, cervix and colorectal and for self-reported family history were cancers of the gastrointestinal tract, pulmonary, breast, head/neck and gynecological system. Common associations were with first degree relatives (single parent affected 50.8%, both parents affected 1.8%, siblings affected 21.9% and parents and siblings affected 3.1%). Involvement of grandparents accounted for 13.4%. The numbers affected ranged from one to three family members. For self-reported personal history of cancers, older age and gender were significant on univariate analysis and remained on multivariate analyses (p<0.05). For self–reported family history of cancers, older age, gender, professionals employment and smoking status were significant on univariate analysis but only older age, gender, race and professional employments remained significant factors on multivariate analyses (p<0.05).

**Conclusions:**

Our study showed that more than one in nine of participants reported personal or family histories of cancers, and certain characteristics were predictive of self-reporting history of cancers. Associations of cancers were mainly with first degree relatives.

## Introduction

Worldwide, overall cancer incidence is increasing and represent a major cause of morbidity and mortality. It is also a major drain on the healthcare resources. In the more developed nations, the incidences of some cancer have plateaued or even started to decline (IARC, GLOBOCAN 2012). On the other hand, the incidence continue to increase in developing nations, especially cancers that are associated with changing demographics. These increases have been attributed to the ageing population (Jemal et al., 2011; Miller et al., 2016) and to some extend change in lifestyle, in particular sedentary lifestyles (Stein and Colditz; 2004; Redeker et al., 2009; Al-Azri et al., 2014; Lagerlund et al., 2015). 

Data on cancer incidence have largely come from cancer registry studies and such studies provide information of the incidence and trending of cancers. However, such studies do not provide information on how many people are affected and their relation to those affected with cancers. Such information will be useful when discussing with our patients. To date, there have been only few studies looking at the incidence of self-reported cancers, either personal or family history of cancers. Doubts remain on the reliability of self-reported cancers (Navarro et al., 2006). However, many studies have reported acceptable to good reliability (Stavrou et al., 2011; Fiederling et al., 2016) of self-reporting on cancers. The overall sensitivity of self-reporting on cancers vary from as low as 57.5% (Navarro et al., 2006) to as high of >90% (Stavrou et al., 2011; Fiederling et al., 2016), but dependent of variable factors. Generally, self-reported cancers is sufficiently valid to be used for epidemiological studies but need to take into account the type of cancers and patients or subjects demographic. Reliability of self-reported medical conditions has also been shown for other diseases such as myocardial infarction, stroke, diabetes mellitus, and hypertension (Okura et al., 2014; Machón et al., 2013). Similar to studies on self-reported cancers, validity is not seen for all inquired conditions. 

To date, there is no data on self-reported incidence of cancers in our setting. The aims of this study are to report on the incidence and types of self-reported personal history and family history of cancers among civil servants who participated in a large health survey (Integrated Health Screening Survey) in Brunei Darussalam and predictive factors of self-reported cancers. 

## Materials and Methods

Patient Population: This study was a retrospective analysis of data collected from a health screening study (Integrated Health Screening Study). During the period from 2008 to 2013, civil servants were invited to participate in this health screening study which assessed multiple parameters including data on personal and family history of cancers. All civil servants during the study period were invited through cooperation with the various ministries, and participations where voluntarily. Selected participants were invited through schedule appointment slots for assessment and also given the questionnaire to complete. They were given a period to return the questionnaires.

In this screening survey, a detailed questionnaire was used to collect data on demographic, comorbid conditions, family history, lifestyle and also investigations for screening of common Non-communicable diseases (NCDs) such as hypertension, dyslipidemia, diabetes mellitus and ischemic heart disease. Participants reporting any personal or family history cancers were required to provide additional details; a) types of cancers, b) person/s affected (self, family or both or others), and c) associations (first degrees: parents, siblings and children; second degrees; uncles, aunties, cousins or grandparents or c) others who are of no blood relations), and the numbers affected with cancers. 

Data collected were entered in the Microsoft Excel database and identifying details were removed for anonymity. The study was conduct following the recommendation of the Declaration of Helsinki.

*Definitions*

In our setting, employment status was categorized into different divisions and this correlates with expertise and pay-scale grades. Employment is divided into five divisions; Division I - professionals with the highest range pay grade (i.e. Directors and above, doctors with higher professional qualifications, higher educational institution lecturers etc…); II – Professionals (i.e. Doctors without higher professional qualification, education officers, etc…) ; III – Assistants, Clericals etc…, IV – Clerical and non-professionals; and V – non-professional laborers.

*Categorizations*

History of cancers was categorized into self-reported personal history and self-reported family history. The associations were categorized into first degree relations (parents, siblings and children), second degree relations (grandparents, uncles, aunties and cousins) and others (no blood relations; non-relatives and friends). For family history of cancer, the types of cancers were categorized into systems; gastrointestinal tract, pulmonary, head and neck, skin and soft tissue, gynecological, urology, male reproductive organ, breast, hematological, central nervous system, bones and others. For personal history of cancer, the types of cancers were reported as specific cancers as the subjects were more likely to remember the specific of cancers. 

*Statistical analyses*

For statistical analyses, several multiple variables were grouped into separate categorical variables for univariate and multivariate analyses. The age group be categorized into <40.6 years old vs. above (>40.6 years old) based on the median age of the participants, gender, racial groups into Malays vs. others (Chinese, Indigens and expatriates), marital status (single vs. married/separated/divorced), employment divisions (Divisions I/II vs. III/IV/V), comorbid conditions (presence or absence) and smoking status (active or past smoker vs. non-smoker). 

For comparative analyses, those without details of either personal of family history of cancers were excluded from analyses. Descriptive statistics were used and data were presented as absolute number and percentage. Continuous variables were presented as mean and standard deviation. Multiple continuous variables were compared with ANOVA with Bonferroni post hoc analysis. For univariate analyses, the Chi-Square test was used to compare the categorical variables. A p value of less than 0.05 on univariate analyses was taken as significant and entered in logistic linear regression multivariate analyses to assess for variables predictive of self-reporting of personal and family history of cancers.

## Results

There were a total of 49,760 civil servants who were invited to participate in this Integrated Health Questionnaire study and 21,437 subjects who completed the questionnaire given a response rate of 43.1%. The baseline demographic of the subjects is shown in Table 1. 

Overall, self-reported history of cancers was 11.2% (n=2,408); personal history (n=137, 0.6%) and family history (n=2,021 9.4%) with a proportion (n=250, 1.2%) reported history of cancer but did not specify whether personal or family history. Only 0.1% (n=25) of participants reported both personal and family history of cancers. More female participants reported personal and family history of cancers. Among the races, being Chinese was associated with higher incidence of family history of cancer. Among those with self-reported personal history of cancers, the most common cancers were cancer of the breast (n=35), cervix (n=19) and colorectal (n=6) (Table 2). Among those with self-reported family history of cancers, the most common cancers were cancers affecting the gastrointestinal tract, pulmonary, breast, head and neck and gynecological system (Table 3). 

Among those self-reported family history of cancers, common associations were parents only (one parent 50.8%, both parents 1.8%), siblings only (21.9%) and parents and siblings (3.1%). Involvement of grandparents accounted for 13.4%. The numbers affected ranged from one to three family members. 

Table 4 shows the associations of those reported to have cancers. Participants with history of children and history of sibling/s with cancers were significantly older (p<0.05, ANOVA with Bonferroni post hoc analysis) (Table 5). 

For self-reported personal history of cancer; older age and being female were significant on univariate analyses (all p values <0.05). Active/past smoker was just outside of statistical significance. For self-reported family history of cancer; older age, being female, Malay ethnicity, employment division (Divisions I/II-being professionals) and active/past smokers were significant on univariate analyses (all p values <0.05). Marital status was just outside of statistical significance. Factors associated with self-reported of personal and family history of cancers are shown in Table 6.

For self-reported personal history of cancer, older age and being female remained significant on multivariate analysis. For self-reported family history of cancers, older, being female, ethnicities and employment division (Divisions I/II-being professionals) remained significant. Multivariate analyses are shown in Table 7. 

**Table 1:** Demographic of Participants of the Health Screening (N=21,437)

Variable	
Mean age (years)	40.61 ± 9.46
Gender	
Male	9,660 (45.1)
Female	11,777 (54.9)
Race	
Malays	19,803 (92.4)
Chinese	612 (2.9)
Indigenous	19 (0.1)
Others	891 (4.2)
Not stated	112 (0.5)
Marital status	
Single	4,385 (20.5)
Married	16,114 (75.2)
Separated/divorced	426 (2.0)
Not stated	512 (2.4)
Employment division	
I/II	3,555 (16.6)
III/IV	11,366 (53.0)
V	5,301 (24.7)
Not stated	1,215 (5.7%)

Employment division (Divisions I/II, professionals; III/IV, clerical and V, laborer)

**Table 2:** Types of Cancers among Those with Self-Reported Personal History of Cancers (n=137)

Types of cancers	n (%)
Breast	35 (25.5)
Others*	43 (31.4)
Cervix	19 (13.9)
Colorectal cancer	6 (4.4)
Lung	7 (5.1)
Ovarian	1 (0.7)
Not stated **	26 (18.2)

*, Other types of cancers were other cancers not specifically listed above; **, Participant reported personal history of cancer but did not state the types of cancers

**Table 3:** Types of Cancers among Those with Self-Reported Family History of Cancers (N=2,021)

Types of cancers	n (%)
Gastrointestinal tract	431 (21.3)
Pulmonary	304 (15.0)
Breast	241 (11.9)
Head and neck	143 (7.1)
Gynecological	140 (6.9)
Hematological	77 (3.8)
Nervous system	44 (2.2)
Prostate	35 (1.7)
Musculoskeletal	34 (1.7)
Endocrine	20 (1.0)
Urological	16 (0.8)
Dermatological/soft tissue	15 (0.7)
Not stated types of cancer *	521 (26.8)

*participants reported history of cancer but did not specify the types of cancers

**Table 4:** Associations of Those with Reported History of Cancers

Association	n (%)
Personal history of cancers	137 (0.6)
Family history of cancers	2,021 (9.4)
First degree	1,742 (8.1)
Parents	1,206 (5.6)
Father	615 (2.9)
Mother	554 (2.6)
Both parents	37 (0.2)
Siblings	505 (2.4)
Sister (s)	401 (1.9)
Brother (s)	104 (0.5)
Children	31 (0.1)
Second degree	361 (1.7%)
Grandparent (s)	271 (1.3)
Grandfather	101 (0.5)
Grandmother	118 (0.6)
Both grandparents	6 (0.03)
Not stated	46 (0.2)

**Table 5:** Comparisons between the Ages of the Subjects (N=21,187¶)

Associations (those affected)	n	Mean age
Parent	1,063	41.81 ± 8.54
Parent (s) and sibling (s)	63	43.60 ± 7.17 *
Parent (s) and grandparent (s)	10	34.60 ± 7.60
Sibling (s)	443	43.78 ± 8.25 *
Sibling (s) and grandparent (s)	4	37.25 ± 8.62
Grandparent (s)	256	32.79 ± 7.23
Children affected	31	47.64 ± 7.41 *
Not stated (relationships)	151	40.34 ± 9.26
No history self-reported cancer	19,029	40.56 ± 9.50

*, ANOVA significant between siblings, grandparents, children (p<0.05); ¶, excluding participants (n=250) who reported history of cancers but did not report whether it was personal or family history of cancer.

**Table 6:** Variables Predictive of Reporting Personal and Family History of Cancers (Univariate Analyses)

Variables	Personal history		p value	Family history		p value
	Yes	No		Yes	No	
Age						
<40.6 yrs old	35 (25.5)	9,971 (48.2)	<0.001	923 (45.7)	9,330 (48.1)	0.042
≥40.6	102 (74.5)	10,732 (51.8)		1,096 (54.3)	10,069 (51.9)	
Gender						
Male	34 (24.8)	9,318 (45.0)	<0.001	708 (35.0)	8.952 (46.1)	<0.001
Female	103 (75.2)	11,403 (55.0)		1,313 (65.0)	10,464 (53.9)	
Races						
Malay	127 (92.7)	19,143 (92.5)	0.926	1,782 (88.4)	18,021 (93.0)	<0.001
Others	10 (7.3)	1,554 (7.5)			233 (11.6)	1,364 (7.0)
Marital status						
Single	20 (15.2)	4,266 (21.8)	0.099	384 (19.3)	4,001 (21.1)	0.061
Married/	112 (84.8)	16,033 (79.0)		1,603 (80.7)	14,937 (78.9)	
Separated/widowed						
Divisions						
I/II	20 (15.2)	3,447 (17.6)	0.467	536 (27.7)	3,019 (16.5)	<0.001
III/IV/V	112 (84.8)	16,175 (82.4)		1,401 (72.3)	15,266 (83.5)	
Comorbid conditions *						
Present	43 (31.9)	5,810 (28.3)	0.367	561 (27.9)	5,454 (28.1)	0.659
Absent	92 (68.1)	14,692 (71.7)		1,447 (72.1)	13,747 (71.6)	
Smoking Status						
Active/exsmoker	19 (13.9)	4,250 (20.5)	0.055	348 (17.2)	4,029 (20.8)	<0.001
Nonsmoker	118 (86.1)	16,471 (79.5)		1,673 (82.8)	15,387 (79.2)	

*, Include some missing data

**Table 7:** Multivariate Analyses for Factors Predictive for Self-Reported History of Any Cancers

	Odd ratio	p value	95% Confidence interval
Personal history			
Age	2.813	<0.001	1.914 to 4.136
Gender	2.583	<0.001	1.751 to 3.812
Family history of any cancer			
Age	1.142	0.006	1.038 to 1.256
Gender	1.671	<0.001	1.491 to 1.873
Race	1.641	<0.001	1.409 to 1.910
Division	0.526	<0.001	0.472 to 0.586

## Discussion

In our study, the overall incidence of self-reported history of cancers was 11.2% representing more than one in nine government servants reporting either a personal or family history of cancers. Of this, a larger proportion reported a family history of cancers (9.4%) and a smaller proportion reporting a personal history of cancer (0.6%). Both personal and family history of cancers were reported by 0.1%. Of those who reported cancers, 1.1% did not state whether the cancers were personal or family history and this, either they could not recall and not sure of the detail. In our study the participants were active government servants with a majority being of Malay ethnicity and a median age of 40.6 years old. This is in contrast to the national demographic makeup where the Malay populations accounts for approximately ~68% of the whole populations. This needs to be considered and our findings may not be considered representative of the whole population but will serve as a useful guide when discussion about matters related to cancers.

The types of cancers reported in our study either personal (top five; cancers of the breast, others systems, cervix, colon-rectum and pulmonary) or family history (top five; gastrointestinal, pulmonary, breast, head and neck and gynecological cancers) although slight differences are consistent with what have been reported in studies based on our cancer registries. On self-reported personal history of cancers, the spectrum is consistent with the fact that more female reported a personal history of cancer. For self-reported family history of cancers, the most common cancers were cancers of the gastrointestinal tract with colorectal cancer (21.3%) being the most common, consistent with the fact that colorectal cancer is one the most common gastrointestinal tract cancers in our country (Mohammed et al., 2014; Ong et al., 2018). The other common cancers are cancers of the pulmonary (15.0%), breast (11.9%), head and neck (7.1%) and gynecological (6.9%) system; cancers that are common in our country (Mohammed et al., 2014; Ong et al., 2018). 

The types of cancer reported in our study are consistent with finding of a large study in the United States. In this study, the most common self-reported cancers were cancers of the breast (11.8%), colon-rectum (9.4%), lung (10.1%) and prostate (7.3%) (Pinsky et al., 2011). Unlike the study by Pinsky et al, prostate cancer was less common in our study accounting for only 1.7%. Our low number is again consistent with cancer spectrum in our country (Ong et al., 2018). Generally the types of cancers reported should also reflect the types of cancers common to the countries. 

Among those who reported a family history of cancers, most reported involvements of first-degree relatives with second-degree relatives involvement almost exclusively grandparents. Among first-degree relatives involvement, parents accounted for the most (62.3%). Fathers were affected slightly more than mothers with a very small proportion of both parents being affected. In our setting, cancers are reported more in men; albeit just slightly more (Mohammed et al., 2014). Among siblings been affected by cancers (25%) in the present study, the most common association was sisters, almost four times more female siblings being affected with cancers. This finding can be explained by the fact that our study population were young and cancers affecting females are commonly diagnosed younger than other cancers; breast (majority in the fourth and fifth decades) and gynecological cancer (i.e. cervical cancers between third and fifth decades) (Leong et al., 2019; Ong et al., 2018). Self-reported history of cancers in offspring or children having cancers only accounted for a small proportion of 0.1%, and this is not unexpected given the age demographic of our participants. 

Participants who reported cancers amongst siblings and children were significantly older compared to those who reported cancers among others. There was no significant differences between those who reported no personal or family history of cancers compared those reporting cancers amongst parents. The youngest were those who reported cancers amongst grandparents and oldest amongst those reported cancers in children. These finding are likely explained by the age range of our study populations with a median age of 40.6 years, all younger than the age of 60 years old, the official retirement age in the government services.

In this study, we showed that several demographic factors were predictive of self-reporting of cancer. For self-reported personal history of cancer, we showed that being older (>40.6 years old) and being female were significant predictive factors. For self-reported family history of cancers, being older, female gender, Malay ethnicity and employment status (being professionals) were all significant factors. Higher incidence of self-reported family history of cancer is not unexpected. Incidence of cancers increases with age and older age meant that the participants would themselves have their own risk of cancers increased and also they would have also relatives of older ages. This is also compounded by the fact that cancer of the young is common in our country (Mohammed et al., 2014). Higher incidence of self-reporting of cancers among female participants, both personal and family history is again reflected by the cancer spectrum in our country. The top three most common cancers amongst women are cancers of the breast, colon-rectum and cervix (Ong et al., 2018). Certain ethnicities or races are well known to be at higher risk of cancers and this also the case in our country (IARC, GLOBOCAN, 2012; Mohammad et al., 2014). In our local contacts, Chinese have higher incidence of colorectal and gastric cancers (Chong et al., 2009; Chong et al., 2014). Association of higher incidence of self-reported history of cancers and being professionals (Division I/II- being professionals) may be due to better awareness and standard of living. It is well known that some of the cancers such as colorectal and breast cancers may be related to improvement of standard of living as reflected by the increasing incidence in some of the developing nations (IARC GLOBOCAN, 2012). 

Unlike self-reported personal history of cancers, there have been debates about the validity of self-reported family history of cancers. There are concerns that the information provided may not be reliable and inaccurate. Studies have shown that the reliability of self-reported family history of cancer is not consistent. However a meta-analysis of 14 cohort studies (Murff et al, 2004) showed that self-reported history is reliable but only for breast and colorectal cancers and only for first degree relatives. The quality for other cancers and second degree relatives are more variable. Colorectal and breast cancers accounted for large proportion of reported cancers. A limitation with our study, being as questionnaire based study was subjected to recall biases and issue of incomplete questionnaires. In our study, a small proportion of participants failed to include information on whether the self-reported cancers were personal or family history of cancers, types of cancers and also in some demographic information. 

In any healthcare settings, it is particularly important to be aware of the epidemiology and the disease spectrum for healthcare and policy planning. Such information is available from cancer registries; however, cancer registries often do not include data on family history of cancers and hence do no inform the clinicians of the proportion of their patients or population who have family members affected with cancers. Such knowledge will allow clinicians to know the magnitude of the cancer epidemic and also provide risk assessments. Therefore our data will be useful as baseline data for healthcare planning and future comparisons. Our cancer spectrums reported by participants corresponds to the cancer spectrums in our local setting and hence our findings can be considered reliable.

There are several limitations with our study that needs to be considered. First; questionnaire studies are inherently associated with recall biases and incomplete data. Second; the study population was active government servants who are younger than 60 years old, the current retirement age. Furthermore, the majority of the study population is of the Malay ethnicity. Hence our study population is not representative of the whole population. However, spectrum of cancers reported for personal and family history are consistent with what is reported in our cancer registry studies and the minor differences accounted for by the findings of more female reporting any history of cancers. Importantly, the sample size of the study is large which an important strength of our study.

In conclusion, our study showed that more than one in nine of the study participants reported history of cancers with the majority reporting a family history of cancers and a much smaller proportion reporting personal history of cancer. The most commonly affected were first degree relatives in particular parents and sibling. The most common secondary degree relatives were grandparents. Female participants reported more family history of cancers. The spectrum of self-reported cancers was consistent with what have been reported with gastrointestinal cancers being the most common, followed by breast and pulmonary cancers. 
